# Statistical Uncertainties of Space Plasma Properties Described by Kappa Distributions

**DOI:** 10.3390/e22050541

**Published:** 2020-05-13

**Authors:** Georgios Nicolaou, George Livadiotis

**Affiliations:** 1 Department of Space and Climate Physics, Mullard Space Science Laboratory, University College London, Dorking, Surrey RH5 6NT, UK; 2 Southwest Research Institute, San Antonio, TX 78238, USA; george.livadiotis@swri.org

**Keywords:** kappa distributions, plasmas, methods

## Abstract

The velocities of space plasma particles often follow kappa distribution functions, which have characteristic high energy *tails*. The *tails* of these distributions are associated with low particle flux and, therefore, it is challenging to precisely resolve them in plasma measurements. On the other hand, the accurate determination of kappa distribution functions within a broad range of energies is crucial for the understanding of physical mechanisms. Standard analyses of the plasma observations determine the plasma bulk parameters from the statistical moments of the underlined distribution. It is important, however, to also quantify the uncertainties of the derived plasma bulk parameters, which determine the confidence level of scientific conclusions. We investigate the determination of the plasma bulk parameters from observations by an ideal electrostatic analyzer. We derive simple formulas to estimate the statistical uncertainties of the calculated bulk parameters. We then use the forward modelling method to simulate plasma observations by a typical top-hat electrostatic analyzer. We analyze the simulated observations in order to derive the plasma bulk parameters and their uncertainties. Our simulations validate our simplified formulas. We further examine the statistical errors of the plasma bulk parameters for several shapes of the plasma velocity distribution function.

## 1. Introduction

Scientists study numerous physical mechanisms in space plasmas through the velocity distribution functions (VDFs) of the space plasma particles. State-of-the-art plasma instruments on board space missions are designed to construct the VDFs of the plasma species. Electrostatic analyzers are widely used to resolve the three dimensional (3D) VDFs of ions and electrons in numerous plasma regimes, such as the solar wind (e.g., [1]), the magnetosphere of Venus (e.g., [2]), Earth (e.g., [3,4]), Mars (e.g., [5]), Jupiter (e.g., [6]), Saturn (e.g., [7]), in the vicinity of comets (e.g., [8]), and more. Some of these designs analyze all the plasma species within the sampled energy range together, while others have advanced designs that allow mass composition measurements. The appropriate analysis of the observed VDFs derives the plasma bulk properties that we need to study the plasma dynamics. For example, the statistical moments of the observed VDFs determine the plasma density, bulk velocity, plasma pressure, energy flux, and many other useful physical quantities. The accuracy of the plasma bulk parameters depends on the quality of the plasma particle measurements and the method we use to analyze them.

Space plasma particles usually undergo limited collisions and their velocities do not follow the classic Maxwellian distribution functions. Instead, the VDFs of plasma particles are often kappa distribution functions (e.g., [9,10,11,12,13,14] and references therein). For example, the studies by [15,16,17,18,19,20,21,22] use kappa distribution functions to describe the solar wind plasma species within the heliosphere. Additionally, the studies by [23,24,25,26,27,28,29,30,31] determine kappa VDFs within planetary magnetospheres, and [32] use kappa distributions to describe the plasma electrons within the coma of 67P Churyumov-Gerasimenko. Moreover, the authors of [33,34,35,36,37,38,39] derive the VDFs of plasma particles within the outer heliosphere and within the inner heliosheath and show that kappa distribution functions fit accurately the observations. However, the study by [40] shows that a single kappa distribution cannot describe the entire energy spectra of heliosheath particles within a few eVs to MeVs. Possibly, a combination of kappa distributions is a better description of these observations.

Kappa distributions have characteristic high energy *tails*, which consist of plasma particles with energies well beyond the bulk energy of the plasma population. The shape of such a *tail* is governed by the kappa index of the distribution function, which is a crucial parameter in understanding the kinetic and dynamic properties of the plasma. Kappa distributions arise naturally from Tsallis statistical mechanics [41,42,43], and the value of the kappa index indicates the correlations between the plasma particles and defines the thermodynamic distance of the plasma from the classic thermal equilibrium [44,45]. The thermodynamic origin of kappa distributions is extensively discussed by [46], while the study by [47] argues for the natural connection between the kappa index of the plasma VDFs and the polytropic index, which describes the transition of the plasma from one thermodynamic state to another, and is necessary for the fluid description on the plasma (e.g., [48,49,50,51,52,53,54,55,56,57,58,59]). Data-analyses demonstrate and quantify the specific relationship between the two indices in the terrestrial ionosphere [30] and in the solar wind at 1 au [60,61]. Note also, that the analyses by [62] and [63] investigate the dynamics of plasma electrons and energetic ions in Saturn’s magnetosphere, respectively, using both the kappa distributions and the polytropic description.

Numerous studies demonstrate the challenges in accurately determining kappa distribution functions from space plasma measurements (e.g., [64,65,66,67]). Different analysis methods have specific advantages and disadvantages. For instance, the numerical calculation of the statistical moments of the observed velocity distribution function is computationally cheap and does not require assumptions regarding the analytical model of the distribution (e.g., [67,68]). On the other hand, if we choose the right analytical model to describe the plasma particles, a chi-square minimization algorithm or/and a maximum likelihood method (e.g., [68,69]) can provide reliable results even in cases where the velocity distribution function is not fully observed, but their calculations are a bit more complicated and possibly non-suitable for on-board estimations. Nevertheless, regardless of the analysis method, the accurate determination of the plasma bulk parameters requires a good measurement resolution of the distribution, including the high energy *tails* that are often associated with low plasma flux, and thus high statistical uncertainty. The statistical uncertainties of the plasma measurements propagate uncertainties in the derived plasma bulk parameters. The quantification of the propagated errors in the derived plasma properties is important for the validation of scientific results.

The purpose of this study is to investigate the statistical uncertainties of the plasma statistical moments derived from the numerical analysis of observed kappa distribution functions. We derive simplified formulas for the expected statistical uncertainties as functions of the observed signal. We then simulate the response of a typical electrostatic analyzer in typical plasma conditions. We analyze the simulated measurements in order to derive the statistical moments and their uncertainties. The comparison between our simulations and the expected values shows a good agreement. We extend our simulations to estimate the statistical uncertainties of the bulk parameters for distribution functions with different shapes. We finally highlight the importance of such novel estimations in future applications. This paper is organized as follows. In the next section, we formulate simplified expressions for the expected statistical error of the plasma moments and we demonstrate how we simulate and analyze typical plasma observations. In Section 3, we present our simulation results, which we compare with our predictions. Finally, we discuss our findings in Section 4.

## 2. Methods

### 2.1. Numerical Calculation of the Statistical Moments

We investigate the properties of plasmas with their particle velocity $\vec{U}$ following the 3D isotropic kappa distribution function (e.g., [13,14,44]):

(1) $$f(\vec{U})=N{\left[\frac{m}{2\pi (\kappa -3/2)k_{B}T}\right]}^{3/2}\frac{\Gamma (\kappa +1)}{\Gamma (\kappa -1/2)}{\left[1+\frac{m{\left(\vec{U}-\vec{V}\right)}^{2}}{2\left(\kappa -3/2\right)k_{B}T}\right]}^{-\kappa -1},$$

which is defined by the plasma density *N*, bulk velocity $\vec{V}$, temperature *T*, and kappa index *κ*. In Equation (1), *m* is the mass of the plasma particles, *k*_B_ is the Boltzmann constant, and *Γ* is the gamma function. The SI unit of *f* is m^−6^ s^3^, while in many applications, it is reported in cm^−6^ s^3^. Typical electrostatic plasma instruments register the number of plasma particles (counts, *C*) detected per particle speed U, and solid angle, which is determined by the elevation angle Θ and azimuth angle Φ of the particle flow direction. We set a coordinate system in which the particle velocity components are

(2) $$\begin{array}{l} U_{x}=U\cos\Theta \cos\Phi ,\\ U_{y}=U\cos\Theta \sin\Phi ,\\ U_{z}=U\sin\Theta . \end{array}$$

As explicitly shown in [65,66,67], an electrostatic analyzer will approximately measure

(3) $$C_{\exp}(U,\Theta ,\Phi )\approx GU^{4}f(U,\Theta ,\Phi )\Delta \tau ,$$

where Δ*τ* is the acquisition time, defined as the time interval for which the instrument records particles in each U, Θ, and Φ. A simplified determination of the instrument’s geometric factor (in terms of speed resolution) *G* is

(4) $$G=A_{\mathrm{eff}}\frac{\Delta U}{U}\Delta \Theta \Delta \Phi ,$$

where *A*_eff_ is the effective aperture (usually expressed in cm^2^), determined by the physical aperture size and the detection efficiency; $\frac{\Delta U}{U}$ is the speed resolution; while ΔΘ and ΔΦ are the elevation and azimuth resolution respectively. We note that Equation (3) predicts the average counts per U, Θ, and Φ, and thus *C*_exp_ is generally a non-integer number. For our mathematical expressions in this section, we use the average *C*_exp_ values, which we then compare with simulations that account for the counting statistics (for more, see Section 2.3). In typical plasma analyses, we construct the 3D VDF from the observed counts using the inverse of Equation (3):

(5) $$f_{\exp}(U,\Theta ,\Phi )=\frac{C_{\exp}(U,\Theta ,\Phi )}{U^{4}G\Delta \tau }.$$

Note that some spacecraft data-products are reported directly in physical quantities, such as differential directional energy flux *J*_E_, typically in (cm^2^ sr s)^−1^, or particle differential intensity, typically in (cm^2^ sr s keV)^−1^, among others. The constructed *f*_exp_ is never a perfect representation of the actual plasma *f* owing to the limited instrument’s efficiency, energy, and angular range and resolution. However, a novel analysis of the plasma measurements can estimate the statistical moments of the constructed distribution function *f*_exp_. Here, we focus on the first three orders of velocity moments and the first order speed moment of the 3D VDF. The expected 0th order velocity moment gives the plasma number density:

(6) $$N_{\exp}=\sum_{U}\sum_{\Theta }\sum_{\Phi }f_{\exp}(U,\Theta ,\Phi )U^{2}\delta U\cos\Theta \delta \Theta \delta \Phi ,$$

The first order velocity moment determines the integrated particle flux:

(7) $${\vec{F}}_{\exp}=N_{\exp}{\vec{V}}_{\exp}=\sum_{U}\sum_{\Theta }\sum_{\Phi }\vec{U}f_{\exp}(U,\Theta ,\Phi )U^{2}\delta U\cos\Theta \delta \Theta \delta \Phi ,$$

where ${\vec{V}}_{\exp}$ is the expected bulk velocity of the plasma particles. The second order velocity moment determines the thermal pressure:

(8) $$P_{\exp}=N_{\exp}k_{B}T_{\exp}=\frac{1}{3}\sum_{U}\sum_{\Theta }\sum_{\Phi }m{\left(\vec{U}-{\vec{V}}_{\exp}\right)}^{2}f_{\exp}(U,\Theta ,\Phi )U^{2}\delta U\cos\Theta \delta \Theta \delta \Phi ,$$

where *T*_exp_ is the expected plasma temperature. Finally, the first order speed moment (e.g., [14,67]) from which we can determine the kappa index is

(9) $$\begin{array}{l} M_{\exp}^{1}=N_{\exp}{\left(k_{B}T_{\exp}\right)}^{\frac{1}{2}}\frac{2}{\sqrt{\pi }}{\left(\kappa_{\exp}-\frac{3}{2}\right)}^{\frac{1}{2}}\frac{\Gamma \left(\kappa_{\exp}-1\right)}{\Gamma \left(\kappa_{\exp}-\frac{1}{2}\right)}\\ =\sum_{U}\sum_{\Theta }\sum_{\Phi }\sqrt{\frac{m}{2}{\left(\vec{U}-{\vec{V}}_{\exp}\right)}^{2}}f_{\exp}(U,\Theta ,\Phi )U^{2}\delta U\cos\Theta \delta \Theta \delta \Phi . \end{array}$$

In Equations (6)–(9), δU, δΘ, and δΦ denote the distance between consecutive U, Θ, and Φ pixels, respectively, in which the instrument samples the 3D VDF. For simplification, we consider the special case where the distance between consecutive pixels is equal to their individual resolution, so that δU=ΔU, δΘ=ΔΘ, and δΦ=ΔΦ. Then, according to Equations (4) and (5), Equations (6)–(9) simplify to

(10) $$N_{\exp}=\sum_{U}\sum_{\Theta }\sum_{\Phi }\frac{C_{\exp}(U,\Theta ,\Phi )\cos\Theta }{A_{\mathrm{eff}}\Delta \tau U},$$

(11) $${\vec{F}}_{\exp}=\sum_{U}\sum_{\Theta }\sum_{\Phi }\vec{U}\frac{C_{\exp}(U,\Theta ,\Phi )\cos\Theta }{A_{\mathrm{eff}}\Delta \tau U},$$

(12) $$P_{\exp}=\frac{1}{3}\sum_{U}\sum_{\Theta }\sum_{\Phi }m{\left(\vec{U}-\vec{V}\right)}^{2}\frac{C_{\exp}(U,\Theta ,\Phi )\cos\Theta }{A_{\mathrm{eff}}\Delta \tau U},$$

and

(13) $$M_{\exp}^{1}=\sum_{U}\sum_{\Theta }\sum_{\Phi }\sqrt{\frac{m}{2}{\left(\vec{U}-{\vec{V}}_{\exp}\right)}^{2}}\frac{C_{\exp}(U,\Theta ,\Phi )\cos\Theta }{A_{\mathrm{eff}}\Delta \tau U},$$

respectively.

### 2.2. Expected Uncertainties

Here, we minimize the systematic errors of the derived moments by consider an instrument with high energy and angular resolution, able to capture the entire 3D VDF. The remaining source of errors is mainly the statistical uncertainty of the registered counts. Generally, in counting experiments, the number of registered counts follow the Poisson distribution function, and the statistical uncertainty of a measurement *C*_exp_ is $\sigma_{C,\exp}=\sqrt{C_{\exp}}$. We calculate the expected statistical uncertainties of the statistical moments using the error propagation formula. Equations (10)–(13) are summations in all U, Θ, and Φ, for example, the expected density in Equation (10) can be written as $N_{\exp}=\sum \Delta N$, where ΔN is the contribution from each U, Θ, and Φ. Therefore, $\sigma_{N,\exp}^{2}=\sum {\left(\sigma_{\Delta N}\right)}^{2}$, where $\sigma_{\Delta N}=\left(\partial \Delta N/\partial C_{\exp}\right)\sigma_{C,\exp}$, and according to the above,

(14) $$\sigma_{N,exp}=\sqrt{\sum_{U}\sum_{\Theta }\sum_{\Phi }{\left(\frac{\cos\Theta }{A_{\mathrm{eff}}\Delta \tau U}\right)}^{2}C_{\exp}(U,\Theta ,\Phi )}.$$

Similarly,

(15) $$\sigma_{Fi,\mathrm{out}}=\sqrt{\sum_{U}\sum_{\Theta }\sum_{\Phi }{\left(\frac{U_{i}\cos\Theta }{A_{\mathrm{eff}}\Delta \tau U}\right)}^{2}C_{\exp}(U,\Theta ,\Phi )},$$

(16) $$\sigma_{P,\exp}=\frac{1}{3}\sqrt{\sum_{U}\sum_{\Theta }\sum_{\Phi }{\left(\frac{m(\vec{U}-{\vec{V}}_{\exp})\cos\Theta }{A_{\mathrm{eff}}\Delta \tau U}\right)}^{2}C_{\exp}(U,\Theta ,\Phi )}$$

and

(17) $$\sigma_{M^{1},\exp}=\sqrt{\sum_{U}\sum_{\Theta }\sum_{\Phi }\frac{m}{2}{\left(\vec{U}-{\vec{V}}_{\exp}\right)}^{2}{\left(\frac{\cos\Theta }{A_{\mathrm{eff}}\Delta \tau U}\right)}^{2}C_{\exp}(U,\Theta ,\Phi )}$$

The subscript *i* in Equation (15) denotes the component (*x*, *y*, or *z*) of the velocity vector. In Equations (16) and (17), we consider that the statistical uncertainty in the derived bulk velocity ${\vec{V}}_{\exp}$ is small compared with the statistical uncertainty of the registered counts in most of the instrument’s pixels, and thus is neglected for simplicity. In our analysis, we test the accuracy of the above simplified expressions with simulations of plasma measurements.

### 2.3. Simulations

**Figure 1:**
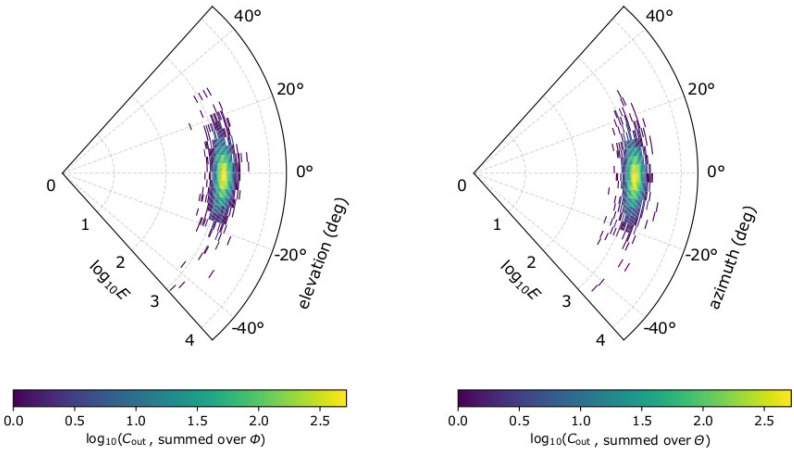
Modeled proton measurements by the electrostatic analyzer design we consider in this study. (**Left**) Number of counts as a function of $\log_{10}Ε$ and elevation angle Θ summed over azimuth Φ; and (**right**) as a function of $\log_{10}Ε$ and Φ, summed over Θ. For the specific example, we consider solar wind protons with N = 20 cm^−3^, $\vec{V}$ = 500 kms^−1^ along the *x*-axis, T = 2 × 10^5^ K, and *κ* = 3.

We use simulations of plasma measurements to validate our formulas in Section 2.1 and Section 2.2. We use the forward modelling method (e.g., [29,64,65,66,67,70,71,72,73,74,75,76,77]) to simulate plasma observations by a typical top-hat electrostatic analyzer with aperture deflectors. The top-hat plane of the instrument model lies in the *x*–*y* plane. The electrostatic analyzer scans the plasma particle energies between 0.2 and 20 keV, in 96 exponentially distributed steps, each with energy acceptance $\frac{\Delta Ε}{Ε}$ of ~5%. This energy range corresponds to proton speeds between 200 kms^−1^ and 2000 kms^−1^, with speed resolution $\frac{\Delta U}{U}$ of ~2.5%. The elevation angle of the flow Θ is measured from the top-hat plane with the use of the aperture deflectors, while the azimuth angle Φ of the flow is resolved on the top-hat plane (measured from x-axis) in discrete azimuth sectors. The modeled instrument resolves both Θ and Φ within −45° to + 45°, in 31 steps with acceptance width ΔΘ = ΔΦ = 3°, respectively. Such a high instrument energy and angular resolution minimizes the systematic errors associated with the poor sampling of the distribution function (e.g., [67]) and allows the investigation of the statistical uncertainties associated with the finite number of registered counts. We also allow the scaling of the instrument’s effective area *A*_eff_ = *K*_eff_*A*_0_ by setting a reference value *A*_0_ = 1 × 10^−6^ m^2^ and varying the *K*_eff_ factor. In order to account for the statistical uncertainty of the registered counts, we simulate plasma measurements following the Poisson counting statistics. More specifically, for a specific plasma f(U,Θ,Φ), we assign a measurement $C_{\mathrm{out}}(U,\Theta ,\Phi )$, randomly selected from the Poisson distribution with an expected (average) value $C_{\exp}(U,\Theta ,\Phi )$ given by Equation (3). In Figure 1, we show the modeled observations by the instrument design with *K*_eff_ = 1, in a test plasma proton population with N = 20 cm^−3^, $\vec{V}$ = 500 kms^−1^ towards the + *x* direction, T = 2 × 10^5^ K, and κ = 3. These plasma parameters will be used through this study as our input test plasma proton population.

Through this paper, we use our simulations to produce plasma measurements for different instrument efficiencies and for different plasma VDFs. For a specific instrument setting and plasma VDF, we model 1000 $C_{\mathrm{out}}(U,\Theta ,\Phi )$ measurement samples. For each sample, we construct 1000 *f*_out_ using Equation (5). We then calculate the statistical moments $N_{\mathrm{out}}$, ${\vec{F}}_{\mathrm{out}}$, $P_{\mathrm{out}}$, and $M_{\mathrm{out}}^{1}$, for each $f_{\mathrm{out}}$, as shown in Equations (10–13). Finally, we compare the mean values and standard deviations of the 1000 derived moments with the corresponding expected values.

## 3. Results

### 3.1. Statistical Moments versus Efficiency

**Figure 2:**
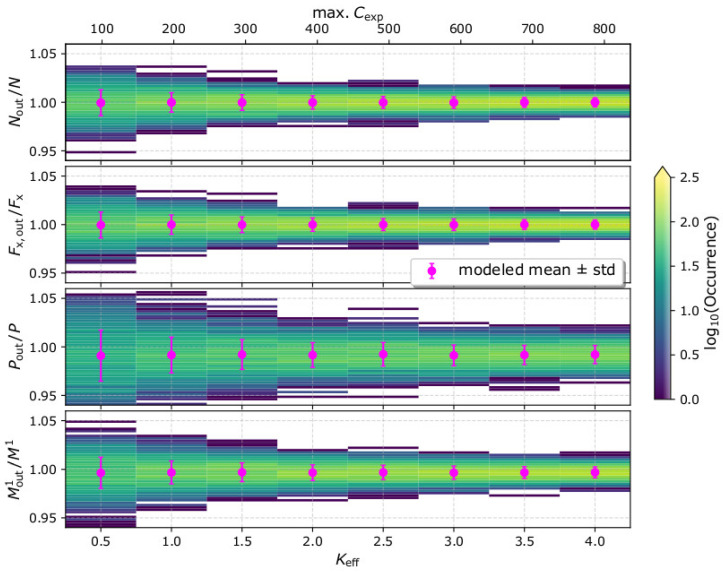
Ratios of the derived plasma distribution moments over the corresponding input moments, as functions of the instrument’s efficiency factor *K*_eff_. For each *K*_eff_ value, we simulate and analyze 1000 measurements samples. (From top to bottom) The derived density (0th order velocity moment) over the input density, the derived *x*-component of the integrated particle flux (first order velocity moment) over the input particle flux, the derived plasma pressure (second order velocity moment) over the input plasma pressure, and the derived first order speed moment over the corresponding input value. The magenta data points show the mean values and standard deviations of the moments in each *K*_eff_ value. The top horizontal axis shows the maximum number of recorded counts max. $C_{\exp}$.

We firstly simulate observations of our test plasma population (see Section 2) for different efficiency factors *K*_eff_. For each *K*_eff_, we analyze 1000 simulated observation samples and calculate $N_{\mathrm{out}}$, ${\vec{F}}_{\mathrm{out}}$, $P_{\mathrm{out}}$, and $M_{\mathrm{out}}^{1}$. In Figure 2, we show the 2D histograms of the derived moments, normalized to their input values, for each *K*_eff_. We show only the *x*-component of the integrated particle flux vector $F_{x,\mathrm{out}}$, which captures the bulk direction of the plasma flow. The relative uncertainty of the derived moments (standard deviation over the corresponding input moment) is significantly small (<3%) and decreases as the instrument’s efficiency and the number of registered counts increase. More specifically, for *K*_eff_ = 0.5, the relative uncertainties of the 0th and the first velocity moments are ~1.5% and drop below 1% for *K*_eff_ > 1.5. The relative uncertainty of the second velocity moment is 2.6% for *K*_eff_ = 0.5, and drops progressively to ~1% for *K*_eff_ = 4. Finally, the relative standard deviation of the first order speed moment is ~1.6% for *K*_eff_ = 0.5 and becomes smaller than 1% for *K*_eff_ > 1.5.

**Figure 3:**
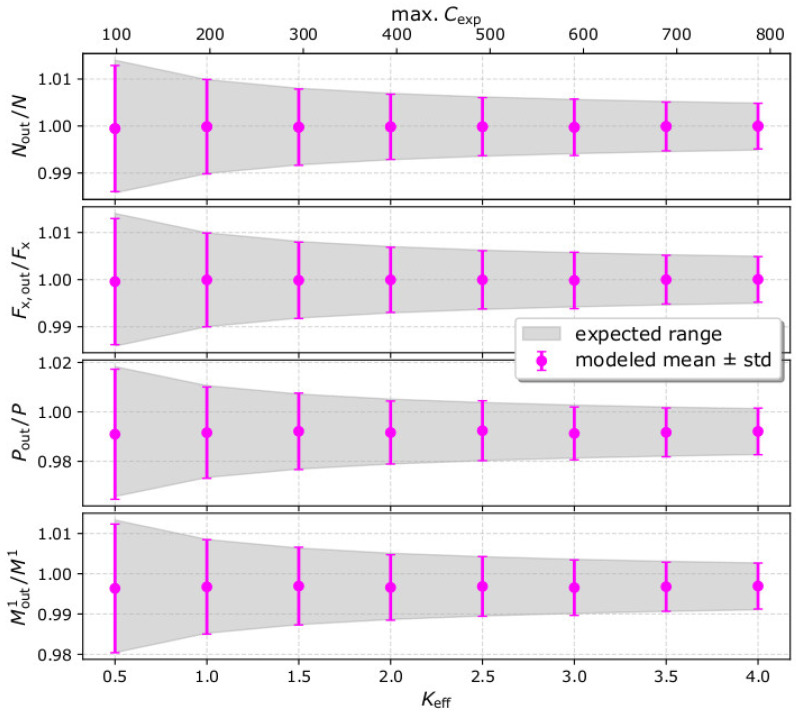
Comparison between the derived (magenta) and the expected range (shadow) of the plasma velocity and speed moments as functions of *K*_eff_. (From top to bottom) The comparisons for the plasma density (0th order velocity moment), the derived *x*-component of the integrated particle flux (first order velocity moment), the plasma pressure (second order velocity moment), and the first order speed moment, while the top horizontal axis shows the maximum number of recorded counts max. $C_{\exp}$. The moments are normalized to the corresponding input moments. The expected range is calculated by Equations (14)–(17) using the expected (average) number of registered counts, while the derived parameters are calculated by the analysis of 1000 measurement samples for each *K*_eff_ value.

In Figure 3, we plot the mean values of the derived moments and their standard deviations as functions of *K*_eff_. The values are normalized to the input moments. In the same plot, we show the corresponding expected values and their uncertainties as determined from the simplified Equations in Section 2. The simulation results are virtually the same as the expected values.

### 3.2. Bulk Parameters versus Kappa Index

**Figure 4:**
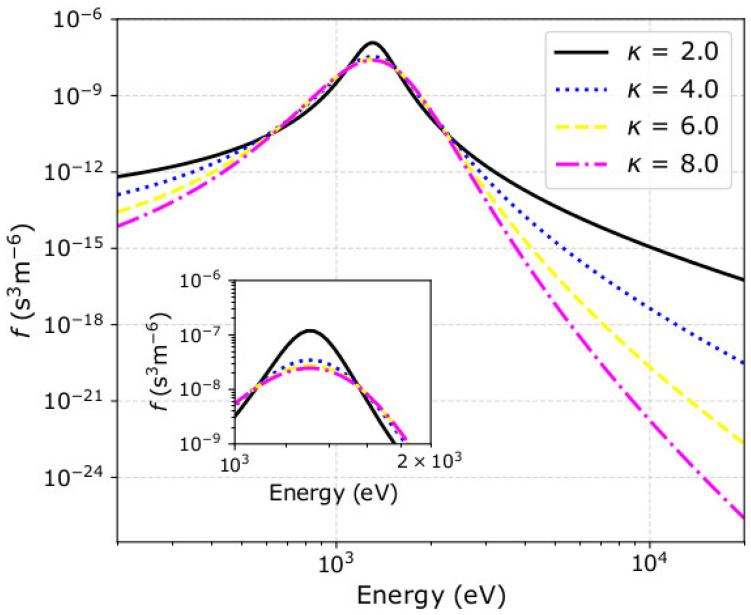
One-dimensional cuts through kappa 3D velocity distribution functions (VDFs) as functions of the particle kinetic energy $E=\frac{1}{2}mU^{2}$. We consider the test proton plasma particles as in Section 2, but for (black solid) κ = 2, (blue dotted) κ = 4, (yellow dashed) κ = 6, and (pink dotted-dashed) κ = 8. The 1D cuts are along the direction of the bulk velocity vector (*x*-axis) and the smaller panel zooms into the *cores* of the VDFs.

The statistical moments are products of the plasma bulk parameters (N, $\vec{V}$, T, and κ) that define the plasma VDF. Previous studies have shown that the accuracy of the derived plasma parameters depends on the shape of the plasma VDF, which is modified by the plasma bulk parameters in a rather complicated way (e.g., [29,66,67,73,74,76]). Here, we use our simulations to investigate the accuracy of the derived bulk parameters as a function of the kappa index, which governs the shape of the plasma VDF. As we show in Figure 4, VDFs with smaller κ have narrower *cores* and their *tails* extend to higher energies.

**Figure 5:**
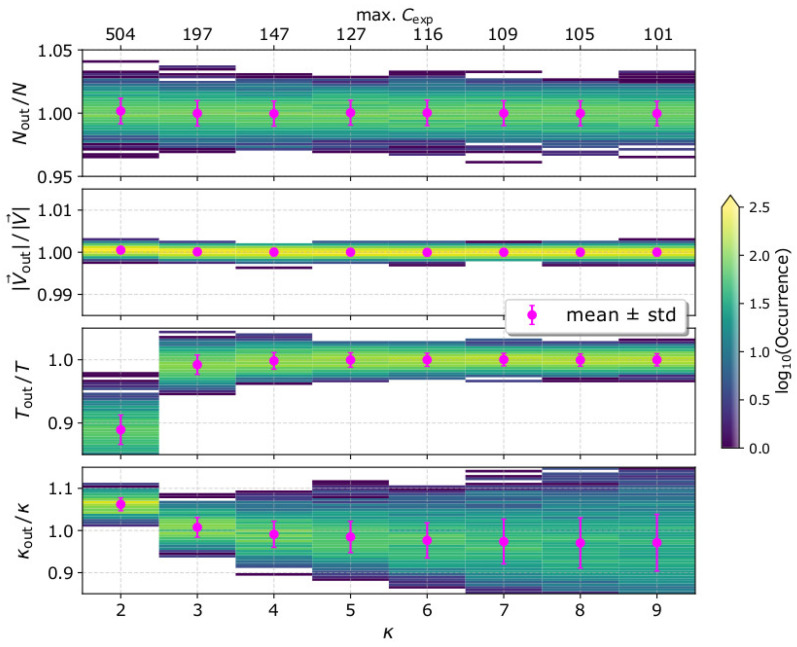
Histograms of the derived bulk parameters $N_{\mathrm{out}}$, $\left|{\vec{V}}_{\mathrm{out}}\right|$, $T_{\mathrm{out}}$, and $\kappa_{\mathrm{out}}$ normalized to their input values, as functions of the input κ. The parameters are derived from the analysis of 1000 measurement samples for each *κ* value. The magenta data points show the mean values and standard deviations of the derived parameters. The top-horizontal axis shows the maximum number of expected counts as a function of the kappa index.

In Figure 5, we show the histograms of the derived bulk parameters $N_{\mathrm{out}}$, $\left|{\vec{V}}_{\mathrm{out}}\right|$, $T_{\mathrm{out}}$, and $\kappa_{\mathrm{out}}$, as functions of the kappa index of the plasma VDF, while in Table 1, we show their mean values and standard deviations. For each kappa index value, we simulate 1000 observation samples by the same plasma sensor (*K*_eff_ = 1). In the top horizontal axis, we show the maximum number of expected counts for each kappa index. For κ = 2, the narrow *core* is associated with ~504 counts. The number of counts drops rapidly to ~197 for a distribution with κ = 3. However, the maximum number of expected counts drops only by a factor of 2 as κ increases from 3 to 9. The relative uncertainty of the derived plasma parameters is significantly small (<3%) for the input plasma parameters and the instrument characteristics we examine here. The relative standard deviation of the plasma density is ~1% and does not change significantly with increasing κ. The relative uncertainty of the plasma speed is extremely small (<1%) and is constant with κ. On the other hand, the standard deviation of the derived temperature decreases from ~2.5% to ~1% as κ increases from 2 to 9. Finally, the standard deviation of the derived kappa index increases with increasing input κ. We note, however, that the accuracy of $\kappa_{\mathrm{out}}$ is much more critical for VDFs with low κ as their elongated *tails* contribute largely to the particle moments (e.g., [12,13,64,78]). In general, a change in the kappa index changes the shape of the VDF and simultaneously affects the number of registered counts, which both affect the accuracy of the plasma parameters, possibly in a different manner. In the next section, we discuss our results in detail.

## 4. Discussion

We formulate simplistic expressions for the statistical moments of the plasma VDFs, as constructed from measurements by typical plasma instruments. We express the statistical moments and their uncertainties as functions of the recorded counts per speed and flow direction, scanned by the instrument C(U,Θ,Φ). Our simplistic expressions show that the statistical moments are proportional to C(U,Θ,Φ), while their statistical uncertainties expected by Poisson counting statistics are proportional to $\sqrt{C(U,\Theta ,\Phi )}$. As a result, the relative uncertainties of the derived moments and the plasma bulk parameters decrease with the increasing number of recorded counts. The analysis of simulated data verifies the simplified equations (Figure 2 and Figure 3). We simulate observations of our test plasma population, and we vary the instrument’s efficiency such as the maximum number of recorded counts increases progressively from ~100 to 800. With the specific settings, the relative uncertainties of all the statistical moments are significantly small and drop below 1% as the maximum recorded counts exceed 300.

Additionally, we quantify the statistical accuracy of the derived plasma density, speed, temperature, and kappa index considering a wide range of input kappa indices, and a specific plasma instrument with a certain efficiency (Figure 5). The kappa index governs the shape of the VDF and the distribution of the recorded counts C(U,Θ,Φ). The narrow *core* of a VDF with a small *κ* is associated with large number of counts limited within a few pixels of the instrument, and the VDF extends to high energies, which contribute mostly to the higher order moments (e.g., Figure 4). With a careful examination of our simplistic expressions in Section 2, we realize that the relative uncertainty of the plasma parameters decreases with increasing counts in individual pixels, and with the increasing number of pixels with detected counts. Therefore, a conclusive examination should quantify the uncertainties as functions of the kappa index and the number of registered counts simultaneously.

**Figure 6:**
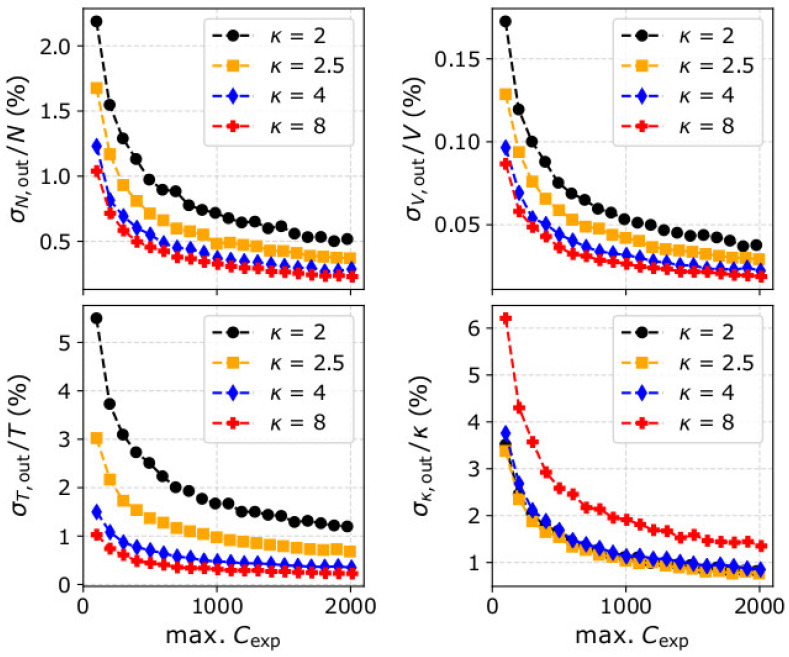
Normalized standard deviations of the derived (upper left) plasma density, (upper right) bulk speed, (lower left) temperature, and (lower right) kappa index as functions of the maximum counts *C*_exp_, and for VDFs with the same density, speed, and temperature, but four different input kappa indices: (black) κ = 2, (orange) κ = 2.5, (blue) κ = 4, and (red) κ = 8. Each data point is the standard deviation of 1000 values determined from the moments of distribution functions constructed from simulated data.

For this purpose, in Figure 6, we show $\sigma_{N,\mathrm{out}}/N$, $\sigma_{V,\mathrm{out}}/V$, $\sigma_{T,\mathrm{out}}/T$, and $\sigma_{\kappa ,\mathrm{out}}/\kappa$ as functions of the maximum recorded counts for four different kappa indices. For the specific quantification, we simulated observations of three different VDFs, all with the same N, $\vec{V}$, and T as in our test plasma protons in previous sections, but different kappa indices. In our simulations, we vary the instrument’s efficiency in order to vary the maximum number of recorded counts. According to Figure 6, for the same number of maximum counts, the relative uncertainty drops with the kappa index for all the plasma bulk parameters. In other words, for measurements with a similar number of counts at their respective peak, the statistical significance of the plasma parameters is greater when derived for more Maxwellian-like VDFs.

Potential use of our simplified formulas can quantify the errors of the statistical moments from a simple numerical analysis of the recorded signal. Our formulas can be very useful for fast and computationally cheap on-board estimations of the errors associated with the derived plasma moments. Moreover, here, we demonstrate the dependence of the statistical error on the shape of kappa distribution functions, which describe numerous physical systems beyond the velocities of plasma particles (e.g., [79,80,81,82]). Future users can apply our methods to quantify the statistical accuracy of kappa distribution moments within a specific parameter range.

Finally, we note that our study examines only the statistical uncertainty of the derived plasma parameters owing to the counting uncertainty, assuming a Poisson model to describe the expected measurements. As we indicate through the paper, this uncertainty is significantly small, although important, for the wide range of parameters we examine here. In typical analyses, the total uncertainty should account for additional sources of statistical and systematic errors, such as spacecraft charging (e.g., [83,84,85]), calibration uncertainties, limited efficiency, and resolution of the instrument (e.g., [67,76]).

## Figures and Tables

**Table 1:** Mean values and standard deviations of the plasma bulk parameters, derived from the simulations of 1000 measurement samples per input kappa index value.

Input *κ*	max. *C*_exp_	*N*_out_ ± *σ_N_*_,out_ (cm^−3^)	*V*_out_ ± *σ*V_,out_ (kms^−1^)	*T*_out_ ± *σ*_T,out_ (×10^5^ K)	*κ*_out_ ± *σ*_κ,out_
2	504	20.00 ± 0.20	500.3 ± 0.4	1.78 ± 0.05	2.12 ± 0.03
3	197	20.00 ± 0.20	500.0 ± 0.4	1.98 ± 0.03	3.02 ± 0.07
4	147	20.00 ± 0.20	500.0 ± 0.4	2.00 ± 0.03	3.96 ± 0.12
5	127	20.00 ± 0.20	500.0 ± 0.4	2.00 ± 0.02	4.92 ± 0.19
6	116	20.00 ± 0.20	500.0 ± 0.4	2.00 ± 0.02	5.86 ± 0.25
7	109	20.00 ± 0.20	500.0 ± 0.4	2.00 ± 0.02	6.82 ± 0.37
8	105	20.00 ± 0.20	500.0 ± 0.4	2.00 ± 0.02	7.76 ± 0.48
9	101	20.00 ± 0.20	500.0 ± 0.4	2.00 ± 0.02	8.74 ± 0.61

## References

[1] Schwenn R., Rosenbauer H., Miggenrieder H. (1975). Das Plasmaexperiment auf Helios (E1). Raumfahrtforschung.

[2] Barabash S., Sauvaud J.-A., Gunell H., Andersson H., Grigoriev A., Brinkfeldt K., Holmstrom M., Lundin R., Yamauchi M., Asamura K. (2007). The Analyser of Space Plasmas and Energetic Atoms (ASPERA-4) for the Venus Express mission. Planet. Space Sci..

[3] Johnstone A.D., Alsop C., Burge S., Carter P.J., Coates A.J., Coker A.J., Fazakerley A.N., Grande M., Gowen R., Gurgiolo C. (1997). peace: A plasma electron and Current experiment. Space Sci. Rev..

[4] Rème H., Bosqued J.M., Sauvaud J.-A., Cros A., Dandouras J., Aoustin C., Bouyssou J., Camus T., Cuvilo J., Martz C. (1997). the cluster ion spectrometry (cis) experiment. Space Sci. Rev..

[5] Barabash S., Lundin R., Andersson H., Brinkfeldt K., Grigoriev A., Gunell H., Holmstrom M., Yamauchi M., Asamura K., Bochsler P. (2007). The Analyzer of Space Plasmas and Energetic Atoms (ASPERA-3) for the Mars Express Mission. Space Sci. Rev..

[6] McComas D.J., Alexander N., Allegrini F., Bagenal F., Beebe C., Clark G., Crary F., Desai M., Santos A.D.L., Demkee D. (2013). The Jovian Auroral Distributions Experiment (JADE) on the Juno Mission to Jupiter. Space Sci. Rev..

[7] Young D.T., Berthelier J.J., Blanc M., Burch J.L., Coates A.J., Goldstein R., Grande M., Hill T.W., Johnson R.E., Kelhä V. (2004). Cassini Plasma Spectrometer Investigation. Space Sci. Rev..

[8] Nilsson H., Lundin R., Lundin K., Barabash S., Borg H., Norberg O., Fedorov A., Sauvaud J.-A., Koskinen H.E.J., Kallio E. (2006). RPC-ICA: The Ion Composition Analyzer of the Rosetta Plasma Consortium. Space Sci. Rev..

[9] Leubner M.P. (2002). A Nonextensive Entropy Approach to Kappa-Distributions. Astrophys. Space Sci..

[10] Leubner M.P. (2004). Fundamental issues on kappa-distributions in space plasmas and interplanetary proton distributions. Phys. Plasmas.

[11] Shizgal B.D. (2007). Suprathermal particle distributions in space physics: Kappa distributions and entropy. Astrophys. Space Sci..

[12] Pierrard V., Lazar M. (2010). Kappa Distributions: Theory and Applications in Space Plasmas. Sol. Phys..

[13] Livadiotis G., McComas D.J. (2013). Understanding Kappa Distributions: A Toolbox for Space Science and Astrophysics. Space Sci. Rev..

[14] Livadiotis G. (2017). Kappa distributions: Theory and Applications in Plasmas.

[15] Maksimović M., Pierrard V., Riley P. (1997). Ulysses electron distributions fitted with Kappa functions. Geophys. Res. Lett..

[16] Maksimović M., Zouganelis I., Chaufray J.-Y., Issautier K., Scime E.E., Littleton J.E., Marsch E., McComas D.J., Salem C., Lin R.P. (2005). Radial evolution of the electron distribution functions in the fast solar wind between 0.3 and 1.5 AU. J. Geophys. Res. Space Phys..

[17] Pierrard V., Maksimovic M., Lemaire J. (1999). Electron velocity distribution functions from the solar wind to the corona. J. Geophys. Res. Space Phys..

[18] Marsch E. (1991). Kinetic Physics of the Solar Wind Plasma. Atmos. Electrodyn..

[19] Zouganelis I., Maksimović M., Meyer-Vernet N., Lamy H., Issautier K. (2004). A Transonic Collisionless Model of the Solar Wind. Astrophys. J..

[20] Štverák Š., Maksimović M., Trávníček P.M., Marsch E., Fazakerley A.N., Scime E.E. (2009). Radial evolution of nonthermal electron populations in the low-latitude solar wind: Helios, Cluster, and Ulysses Observations. J. Geophys. Res. Space Phys..

[21] Yoon P.H. (2014). Electron kappa distribution and quasi-thermal noise. J. Geophys. Res. Space Phys..

[22] Heerikhuisen J., Zirnstein E.J., Pogorelov N.V. (2015). κ-distributed protons in the solar wind and their charge-exchange coupling to energetic hydrogen. J. Geophys. Res. Space Phys..

[23] Leubner M.P. (1982). On Jupiter’s whistler emission. J. Geophys. Res. Space Phys..

[24] ChristoniD S. (1987). A comparison of the Mercury and Earth magnetospheres: Electron measurements and substorm time scales. Icarus.

[25] Leubner M. (2001). Energetic tail evolution of auroral electron spectra. Phys. Chem. Earth, Part C Solar Terr. Planet. Sci..

[26] Mauk B.H., Mitchell D.G., McEntire R.W., Paranicas C.P., Roelof E.C., Williams D.J., Krimigis S.M., Lagg A. (2004). Energetic ion characteristics and neutral gas interactions in Jupiter’s magnetosphere. J. Geophys. Res. Space Phys..

[27] Dialynas K., Krimigis S.M., Mitchell D.G., Hamilton D., Krupp N., Brandt P. (2009). Energetic ion spectral characteristics in the Saturnian magnetosphere using Cassini/MIMI measurements. J. Geophys. Res. Space Phys..

[28] Ogasawara K., Angelopoulos V., Dayeh M., Fuselier S.A., Livadiotis G., McComas D.J., McFadden J.P. (2013). Characterizing the dayside magnetosheath using energetic neutral atoms: IBEX and THEMIS observations. J. Geophys. Res. Space Phys..

[29] Nicolaou G., McComas D.J., Bagenal F., Elliott H. (2014). Properties of plasma ions in the distant Jovian magnetosheath using Solar Wind Around Pluto data on New Horizons. J. Geophys. Res. Space Phys..

[30] Ogasawara K., Livadiotis G., Grubbs G., Michell R., Samara M., Sharber J.R., Winningham J., Jahn J.-M. (2017). Properties of suprathermal electrons associated with discrete auroral arcs. Geophys. Res. Lett..

[31] Kirpichev I.P., Antonova E.E. (2020). Dependencies of Kappa Parameter on the Core Energy of Kappa Distributions and Plasma Parameter in the Case of the Magnetosphere of the Earth. Astrophys. J..

[32] Broiles T., Livadiotis G., Burch J., Chae K., Clark G., Cravens T.E., Davidson R., Eriksson A., Frahm R.A., Fuselier S.A. (2016). Characterizing cometary electrons with kappa distributions. J. Geophys. Res. Space Phys..

[33] Decker R., Krimigis S. (2003). Voyager observations of low-energy ions during solar cycle 23. Adv. Space Res..

[34] Zank G.P., Heerikhuisen J., Pogorelov N.V., Burrows R., McComas D.J. (2009). microstructure of the heliospheric termination shock: Implications for energetic Neutral atom observations. Astrophys. J..

[35] Livadiotis G., McComas D.J., Dayeh M., Funsten H.O., Schwadron N.A. (2011). first sky map of the inner heliosheath temperature usingibexspectra. Astrophys. J..

[36] Livadiotis G., McComas D.J., Randol B., Funsten H.O., Möbius E.S., Schwadron N.A., Dayeh M., Zank G.P., Frisch P.C. (2012). pick-Up ion distributions and their influence on energetic NEUTRAL Atom spectral Curvature. Astrophys. J..

[37] Livadiotis G., McComas D.J., Schwadron N.A., Funsten H.O., Fuselier S.A. (2012). pressure of the proton plasma in the inner heliosheath. Astrophys. J..

[38] Livadiotis G., McComas D.J. (2011). the influence of pick-up ions on space plasma distributions. Astrophys. J..

[39] Livadiotis G., McComas D.J. (2012). non-equilibrium thermodynamic processes: Space plasmas and the inner heliosheath. Astrophys. J..

[40] Dialynas K., Krimigis S.M., Decker R.B., Mitchell D.G. (2019). Plasma Pressures in the Heliosheath From Cassini ENA and Voyager 2 Measurements: Validation by the Voyager 2 Heliopause Crossing. Geophys. Res. Lett..

[41] Tsallis C. (1988). Possible generalization of Boltzmann-Gibbs statistics. J. Stat. Phys..

[42] Tsallis C. (2009). Introduction to Nonextensive Statistical Mechanics: Approaching A Complex World.

[43] Tsallis C., Mendes R., Plastino A. (1998). The role of constraints within generalized nonextensive statistics. Phys. A Stat. Mech. Its Appl..

[44] Livadiotis G., McComas D.J. (2011). invariant kappa distribution in space plasmas out of equilibrium. Astrophys. J..

[45] Livadiotis G. (2015). Kappa and q Indices: Dependence on the Degrees of Freedom. Entropy.

[46] Livadiotis G. (2018). Thermodynamic origin of kappa distributions. EPL (Europhysics Lett.).

[47] Livadiotis G. (2019). On the Origin of Polytropic Behavior in Space and Astrophysical Plasmas. Astrophys. J..

[48] Totten T.L., Freeman J.W., Arya S. (1995). An empirical determination of the polytropic index for the free-streaming solar wind using Helios 1 data. J. Geophys. Res. Space Phys..

[49] Bavassano B., Bruno R., Rosenbauer H. (1996). Compressive fluctuations in the solar wind and their polytropic index. Annal. Geophys..

[50] Newbury J.A., Lindsay G.M., RusselliD C.T. (1997). Solar wind polytropic index in the vicinity of stream interactions. Geophys. Res. Lett..

[51] Kartalev M., Dryer M., Grigorov K., Stoimenova E. (2006). Solar wind polytropic index estimates based on single spacecraft plasma and interplanetary magnetic field measurements. J. Geophys. Res. Space Phys..

[52] Nicolaou G., Livadiotis G., Moussas X. (2013). Long-Term Variability of the Polytropic Index of Solar Wind Protons at 1 AU. Sol. Phys..

[53] Pang X., Cao J., Ma Y. (2016). Polytropic index of magnetosheath ions based on homogeneous MHD Bernoulli Integral. J. Geophys. Res. Space Phys..

[54] Livadiotis G. (2016). superposition of polytropes in the inner heliosheath. Astrophys. J. Suppl. Ser..

[55] Nicolaou G., Livadiotis G. (2017). Modeling the Plasma Flow in the Inner Heliosheath with a Spatially Varying Compression Ratio. Astrophys. J..

[56] Park J.-S., Shue J., Nariyuki Y., Kartalev M. (2019). Dependence of Thermodynamic Processes on Upstream Interplanetary Magnetic Field Conditions for Magnetosheath Ions. J. Geophys. Res. Space Phys..

[57] Elliott H., McComas D.J., Zirnstein E.J., Randol B.M., Delamere P.A., Livadiotis G., Bagenal F., Barnes N.P., Stern S.A., Young L.A. (2019). Slowing of the Solar Wind in the Outer Heliosphere. Astrophys. J..

[58] Verscharen D., Klein K.G., Maruca B.A. (2019). The multi-scale nature of the solar wind. Living Rev. Sol. Phys..

[59] Nicolaou G., Livadiotis G., Wicks R.T. (2019). On the Calculation of the Effective Polytropic Index in Space Plasmas. Entropy.

[60] Livadiotis G. (2018). Using Kappa Distributions to Identify the Potential Energy. J. Geophys. Res. Space Phys..

[61] Nicolaou G., Livadiotis G. (2019). Long-term Correlations of Polytropic Indices with Kappa Distributions in Solar Wind Plasma near 1 au. Astrophys. J..

[62] Arridge C.S., McAndrews H., Jackman C.M., Forsyth C., Walsh A.P., Sittler E., Gilbert L., Lewis G., RusselliD C.T., Coates A.J. (2009). Plasma electrons in Saturn’s magnetotail: Structure, distribution and energisation. Planet. Space Sci..

[63] Dialynas K., Roussos E., Regoli L., Paranicas C.P., Krimigis S.M., Kane M., Mitchell D.G., Hamilton D.C., Krupp N., Carbary J.F. (2018). Energetic Ion Moments and Polytropic Index in Saturn’s Magnetosphere using Cassini/MIMI Measurements: A Simple Model Based on κ-Distribution Functions. J. Geophys. Res..

[64] Nicolaou G., Livadiotis G. (2016). Misestimation of temperature when applying Maxwellian distributions to space plasmas described by kappa distributions. Astrophys. Space Sci..

[65] Nicolaou G., Livadiotis G., Owen C.J., Verscharen D., Wicks R.T. (2018). Determining the Kappa Distributions of Space Plasmas from Observations in a Limited Energy Range. Astrophys. J..

[66] Nicolaou G., Wicks R.T., Livadiotis G., Verscharen D., Owen C., Kataria D. (2020). Determining the Bulk Parameters of Plasma Electrons from Pitch-Angle Distribution Measurements. Entropy.

[67] Nicolaou G., Livadiotis G., Wicks R.T. (2020). On the Determination of Kappa Distribution Functions from Space Plasma Observations. Entropy.

[68] Kasper J.C. (2003). Solar Wind Plasma: Kinetic Properties and MicroInstabilities. Ph.D. Thesis.

[69] Lawless J.F. (2002). Statistical Models and Methods for Lifetime Data.

[70] Elliott H., McComas D.J., Valek P.W., Nicolaou G., Weidner S., Livadiotis G. (2016). The new horizons solar wind around pluto (swap) observations of the solar wind from 11–33 au. Astrophys. J. Suppl. Ser..

[71] Vaivads A., Retinò A., Soucek J., Khotyaintsev Y.V., Valentini F., Escoubet C.P., Alexandrova O., André M., Bale S.D., Balikhin M. (2016). Turbulence Heating ObserveR – satellite mission proposal. J. Plasma Phys..

[72] Cara A., Lavraud B., Fedorov A., De Keyzer J., De Marco R., Marcucci M.F., Valentini F., Servidio S., Bruno R. (2017). Electrostatic analyzer design for solar wind proton measurements with high temporal, energy, and angular resolutions. J. Geophys. Res. Space Phys..

[73] Nicolaou G., McComas D.J., Bagenal F., Elliott H.A., Ebert R.W. (2015). Jupiter’s deep magnetotail boundary layer. Planet. Space Sci..

[74] Nicolaou G., McComas D.J., Bagenal F., Elliott H., Wilson R. (2015). Plasma properties in the deep jovian magnetotail. Planet. Space Sci..

[75] Wilson R.J., Bagenal F., Persoon A. (2017). Survey of thermal plasma ions in Saturn’s magnetosphere utilizing a forward model. J. Geophys. Res. Space Phys..

[76] Nicolaou G., Verscharen D., Wicks R.T., Owen C.J. (2019). The Impact of Turbulent Solar Wind Fluctuations on Solar Orbiter Plasma Proton Measurements. Astrophys. J..

[77] Kim T.K., Ebert R.W., Valek P.W., Allegrini F., McComas D.J., Bagenal F., Chae K., Livadiotis G., Loeffler C.E., Pollock C. (2020). Method to Derive Ion Properties from Juno JADE Including Abundance Estimates for O + and S 2+. J. Geophys. Res. Space Phys..

[78] Livadiotis G. (2019). Theoretical aspects of Hamiltonian kappa distributions. Phys. Scr..

[79] Park J.-S., Jung H.-S. (2002). Modelling Korean extreme rainfall using a Kappa distribution and maximum likelihood estimate. Theor. Appl. Clim..

[80] Ebert R.W., Allegrini F., Fuselier S.A., Nicolaou G., Bedworth P., Sinton S., Trattner K.J. (2014). Angular scattering of 1–50 keV ions through graphene and thin carbon foils: Potential applications for space plasma instrumentation. Rev. Sci. Instruments.

[81] Allegrini F., Ebert R.W., Nicolaou G., Grubbs G. (2015). Semi-empirical relationships for the energy loss and straggling of 1–50 keV hydrogen ions passing through thin carbon foils. Nucl. Instruments Methods Phys. Res. Sect. B Beam Interactions Mater. Atoms.

[82] Kjeldsen T., Ahn H., Prosdocimi I. (2017). On the use of a four-parameter kappa distribution in regional frequency analysis. Hydrol. Sci. J..

[83] Lavraud B., Larson D.E. (2016). Correcting moments of in situ particle distribution functions for spacecraft electrostatic charging. J. Geophys. Res. Space Phys..

[84] Bergman S., Wieser G.S., Wieser M., Johansson F.L., Eriksson A. (2020). The Influence of Spacecraft Charging on Low-Energy Ion Measurements Made by RPC-ICA on Rosetta. J. Geophys. Res. Space Phys..

[85] Voshchepynets A., Barabash S., Ramstad R., Holmström M., Andrews D.J., Nicolaou G., Frahm R., Kopf A., Gurnett D.A. (2018). Ions Accelerated by Sounder-Plasma Interaction as Observed by Mars Express. J. Geophys. Res. Space Phys..

